# Effect of pH on the Emergent Viscoelastic Properties of Cationic Phenylalanine-Derived Supramolecular Hydrogels

**DOI:** 10.3390/gels11110877

**Published:** 2025-11-01

**Authors:** Pamela Agredo, Shruti Ghosh, Brittany L. Abraham, Bradley L. Nilsson

**Affiliations:** 1 Department of Chemistry, University of Rochester, Rochester, NY 14627-0216, USA; pamela.agredosanin@utdallas.edu (P.A.); sghosh18@ur.rochester.edu (S.G.);; 2 Materials Science Program, University of Rochester, Rochester, NY 14627-0166, USA

**Keywords:** supramolecular hydrogels, self-assembly, phenylalanine, viscoelasticity, stimulus-responsive

## Abstract

Supramolecular hydrogels formed by the self-assembly of low-molecular-weight (LMW) agents are promising next-generation biomaterials for drug delivery, tissue engineering, and regenerative medicine. Phenylalanine (Phe) derivatives have emerged as a privileged class of LMW supramolecular gelators due to their strong propensity to self-assemble into emergent hydrogel networks with demonstrated biocompatibility. We have previously reported a series of cationic Phe-derived gelators in which fluorenylmethoxycarbonyl (Fmoc) phenylalanine (Phe), 3-fluorophenylalanine (3F-Phe), and pentafluorophenylalanine (F_5_-Phe) are functionalized at the C-terminus with diaminopropane (DAP). These gelators (Fmoc-Phe-DAP, Fmoc-3F-Phe-DAP, and Fmoc-F_5_-Phe-DAP) are water-soluble and undergo spontaneous self-assembly and gelation upon an increase in the ionic strength of the solution caused by addition of sodium chloride. Herein, we report the effects of pH on the self-assembly and gelation of Fmoc-Phe-DAP, Fmoc-3F-Phe-DAP, and Fmoc-F_5_-Phe-DAP. We also describe the effects that pH has on the emergent properties of these hydrogel networks, including assembly morphology and hydrogel viscoelasticity. These studies indicate that pH has varying effects on the properties of the hydrogels that are also dependent on the molecular structure of the Fmoc-Phe-DAP derivative. Fmoc-Phe-DAP hydrogels are highly sensitive to changes in solvent pH, forming strong hydrogels only near neutral pH. In contrast, hydrogels of Phe derivatives with fluorinated side chains (Fmoc-3F-Phe-DAP and Fmoc-F5-Phe-DAP) have consistent emergent viscoelastic properties across a wider range of acidic to basic pH values.

## 1. Introduction

Hydrogels have emerged as a valuable class of biomaterials with applications in biomedicine that include tissue engineering [1,2,3], regenerative medicine [4,5,6,7], and drug delivery [8,9,10,11,12,13,14]. Chemical hydrogels derived from covalently crosslinked polymers have been widely explored for such applications [15,16,17,18]. However, chemical hydrogels often lack biocompatibility due to immunogenic responses to these synthetic materials [19]. The development of physical hydrogels composed of supramolecular networks of self-assembled materials stabilized by noncovalent interactions have gained attention as attractive alternatives to chemical hydrogels since supramolecular hydrogels are often composed of bioderived, and thus biocompatible, building blocks [14,20]. Biomolecules such as peptides [21,22,23,24,25], nucleobase derivatives [26,27,28,29,30,31], and polysaccharides [32,33,34,35] have been used as building blocks for supramolecular hydrogels.

**Figure 1:**
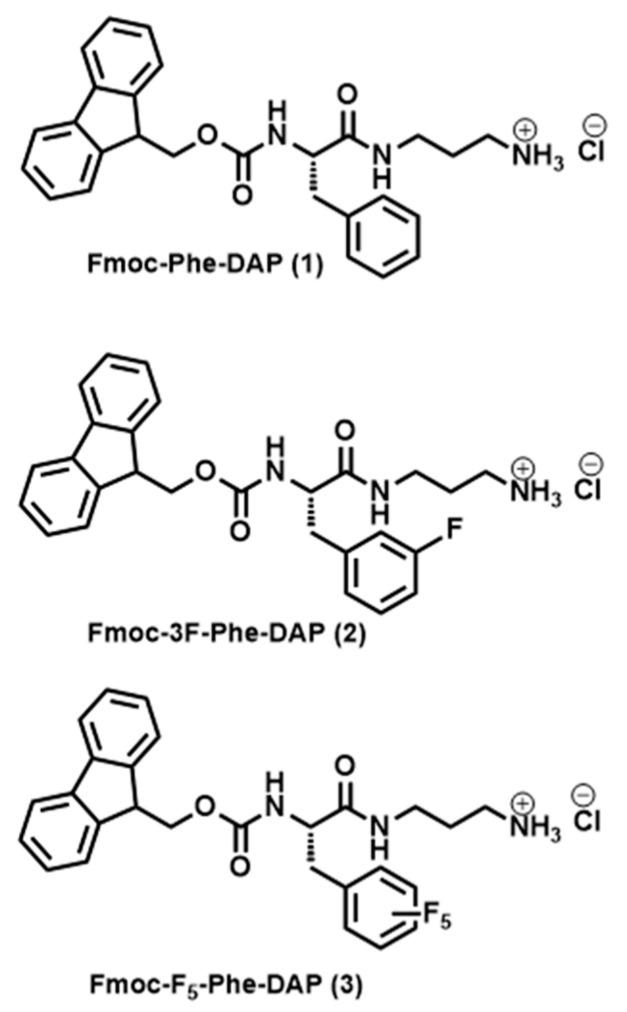
Chemical structures of Fmoc-Phe-DAP derivatives.

Supramolecular peptide-based hydrogels are especially promising as next-generation biomaterials [36]. However, the widespread applications of peptide-based hydrogels have been limited by the high cost of production of synthetic peptides [37]. Therefore, there has been increasing interest in the development of inexpensive, low-molecular-weight (LMW) building blocks, including functionalized amino acid derivatives and dipeptides, as alternatives to synthetic peptides for supramolecular hydrogels [38,39,40,41,42]. Among these amino acid-derived LMW gelators, fluorenylmethoxycarbonyl–phenylalanine (Fmoc-Phe) derivatives have proven to be especially attractive building blocks for supramolecular hydrogels that replicate the desirable viscoelastic and biocompatible properties of peptide-derived counterparts [43,44,45,46,47,48,49]. The self-assembly properties, assembly morphology, and emergent viscoelastic properties of Fmoc-Phe-derived hydrogels are especially sensitive to minute changes in chemical structure, providing strategies to tune these properties as a function of gelator structure [50,51,52]. We have reported a series of cationic Phe-derived gelators that are functionalized with diaminopropane (DAP) at the C-terminus (see Figure 1 for examples) [12,13,53,54,55]. These Fmoc-Phe-DAP derivatives are soluble in water across a broader pH range than the corresponding carboxylic acid derivatives since the p*K*_a_ of the carboxylic acid of the parent Fmoc-Phe molecules is slightly below neutral pH while the p*K*_a_ of the solubilizing ammonium groups of Fmoc-Phe-DAP derivatives is significantly higher than physiological pH, thus allowing hydrogel formulation under a more diverse set of conditions. The sol–gel transition of Fmoc-Phe-DAP derivatives is triggered by increasing the ionic strength of solutions by the addition of physiologically relevant concentrations of sodium chloride or other salts, minimizing charge–charge repulsion that slows self-assembly and weakens hydrogel network interactions. These cationic Fmoc-Phe-DAP hydrogels have been used as drug delivery materials [12,13] and have great potential for a range of other biomedical applications.

The self-assembly and gelation behavior of charged supramolecular gelators is sensitive to the pH of the environment. Supramolecular hydrogels composed of peptides bearing a free carboxylic acid group have been reported to exhibit suppressed ionization leading to significant shifts in carboxyl anion p*K*_a_ related to structural transitions during the self-assembly process [56,57,58,59]. In one example, Adams and coworkers have reported the pH dependent self-assembly behavior of Fmoc–diphenylalanine (Fmoc-FF) hydrogels and have shown that self-assembly results in two apparent p*K*_a_ shifts which are both higher than the theoretical p*K*_a_ for the Fmoc-FF anionic carboxyl group [58]. Further, the network structure and the mechanical properties of these gels change dramatically when the pH of the gels is close to these shifted anion p*K*_a_ values. This and other cited examples indicate the importance of pH as an element of anionic hydrogel design and formulation. As such, it is also expected that pH will have a critical impact on hydrogel formation of cationic charged Fmoc-Phe-DAP derivatives. Accordingly, herein we characterize the effect of pH on the gelation properties of a series of cationic Fmoc-Phe-DAP derivatives, Fmoc-Phe-DAP (**1**), Fmoc-3F-Phe-DAP (**2**), and Fmoc-F_5_-Phe-DAP (**3**). These studies demonstrate that pH impacts the self-assembly properties, assembly morphology, and the emergent viscoelastic properties of supramolecular hydrogels derived from cationic Fmoc-Phe-DAP derivatives. Significantly, these outcomes provide a guideline for exploiting pH in the design and formulation of cationic supramolecular hydrogels.

## 2. Results and Discussion

### 2.1. Self-Assembly and Hydrogelation of Fmoc-Phe-DAP Derivatives from pH 3–10

**Figure 2:**
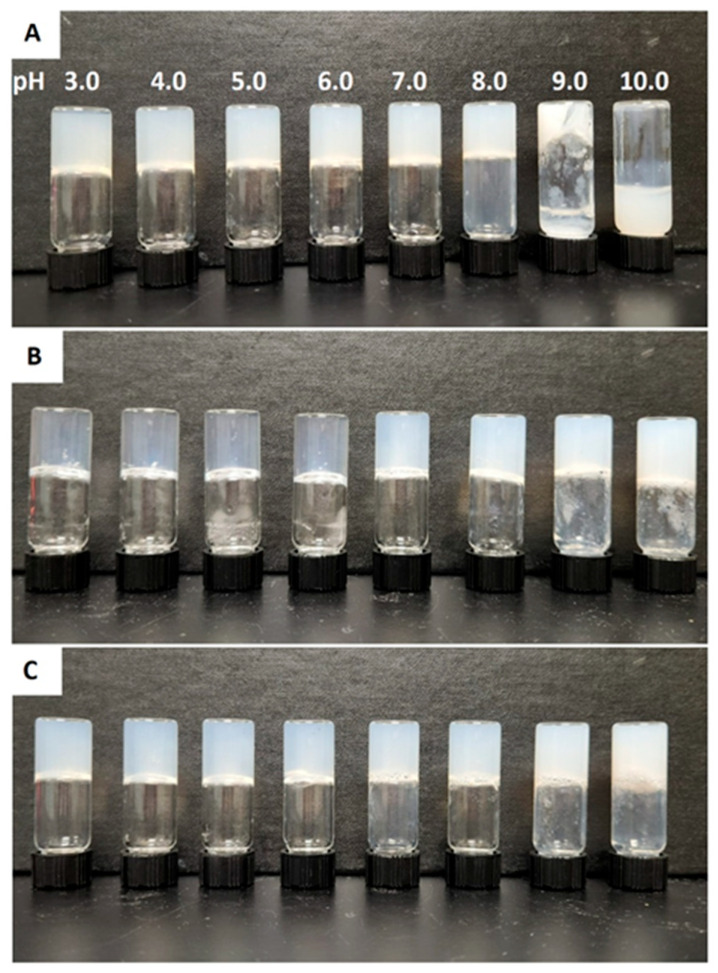
Digital images of (**A**) Fmoc-Phe-DAP (gelator **1**), (**B**) Fmoc-3F-Phe-DAP (gelator **2**), and (**C**) Fmoc-F_5_-Phe-DAP (gelator **3**) gels at pH values ranging from 3.0 to 10.0 after 24 h of gelation initiation.

The gelation properties of three cationic Fmoc-Phe-DAP gelators (compounds **1**–**3**, Figure 1) were characterized over a range of pH values to understand the possible pH dependence on self-assembly and hydrogel formation. The theoretical p*K*_a_ of the DAP ammonium cation is expected to be similar to that of the propylammonium cation which has a reported p*K*_a_ ≈ 10.71 [60,61]. Gels were formulated at 10 mM concentrations of gelators **1**–**3** in glass vials at a final volume of 2 mL. Eight different gels were prepared for each gelator at pH values ranging from 3.0 to 10.0. Significant deprotonation of the DAP ammonium group is expected to happen only at the higher pH ranges (>9.0) based on the theoretical p*K*_a_ of these cations. Gelation was initiated by addition of sodium chloride to a final salt concentration of 114 mM (approximate salt concentration in common cell culture media and physiological saline) to screen ammonium cation charge repulsion that slows self-assembly and network formation [53]. After gelation was triggered, all the gels were allowed to stand for 24 h, and the vials were inverted to determine formation of self-supporting hydrogels as a first approximation (Figure 2). Nearly all samples formed gels across all pH ranges. The resulting gels were more transparent at acidic pH values. As the pH was increased, hydrogels of compound **2** and compound **3** became opaque at neutral or basic pH (7.0 and 9.0 for **2** and **3**, respectively). Gelator **1** fails to form self-supporting hydrogels at high pH values from 9.0 to 10.0, instead forming colloidal suspensions and precipitates. After 2 weeks, these hydrogels underwent some visible changes: gelator **1** precipitates at pH 9.0, near the theoretical p*K*_a_ of the ammonium cation (p*K*_a_ ≈ 10.71) [60,61]. Hydrogels of gelator **2** become more visibly turbid at pH values from 7.0 to 10.0. Gelator **3** precipitates from hydrogels at pH 10.0 and becomes more visibly turbid at pH 7.0 to 9.0 (Figure S1). Each of the gelators forms hydrogels with higher long-term stability over the pH range from 3.0 to 7.0.

### 2.2. Determination of Gelator pK_a_ by Titration

**Figure 3:**
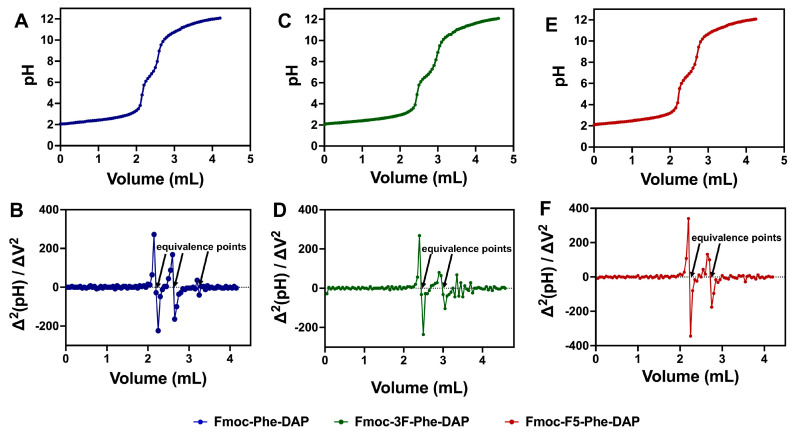
Titration curves of and second-derivative plots, respectively, of (**A**,**B**) gelator **1**, (**C**,**D**) gelator **2**, and (**E**,**F**) gelator **3**.

The ionization behavior of the ammonium DAP cation of gelators **1–3** was characterized by a titration strategy. Samples were dissolved at acidic pH and titrated with NaOH to gradually increase the pH. Plotting pH against the volume of added NaOH enabled the observation of several distinct transitions corresponding to the apparent p*K*_a_ values of the DAP ammonium cations (Figure 3). Solutions of the three gelators (1 mM) were prepared using water at pH 2.0. This was followed by the addition of 50 µL aliquots of 0.1 M NaOH and the pH was measured after each addition. The titration curves for compounds **1**, **2**, and **3** are shown in Figure 3A, Figure 3C, and Figure 3E, respectively. The curve for each compound shows a gradual increase in pH from 2.0 to 3.0, followed by a rapid increase from 3.5 to 6.0 that corresponds to the first equivalence point. A second transition was observed for each compound from pH 8.0–9.0, corresponding to a second equivalence point. A very small third transition was observed between pH 10.5–11, but this could only be clearly defined for Fmoc-Phe-DAP (**1**) hydrogels. At pH 12.0, all solutions presented a white precipitate (Figure S2, Supporting Information), which correlates with complete deprotonation of the gelators at this pH, significantly reducing the water solubility of these compounds. The experimental p*K*_a_ values were determined from these titration measurements by analyzing the second derivative vs. volume plots (Figure 3B,D,F), which were used to identify the titration equivalence points, which were then correlated to the pH vs. volume titration plots (Figure 3A,C,E) to extrapolate the observed p*K*_a_ values.

There were two clear p*K*_a_ values observed in these titration experiments for gelators **1**, **2**, and **3** with a third minor p*K*_a_ value observed for gelator **1** (Table 1). The two initial p*K*_a_ transitions (p*K*_a_ 1 and p*K*_a_ 2) for each gelator are significantly lower than the expected theoretical p*K*_a_ by ~8.0 and ~4.0 pH units, respectively. The first observed p*K*_a_ value (p*K*_a_ 1) was approximately 2.5 for each gelator (Table 1). The second p*K*_a_ value (p*K*_a_ 2) was near neutral pH (6.7–6.8) for each gelator (Table 1). The p*K*_a_ value corresponding to the third transition (p*K*_a_ 3) was only unambiguously observed for gelator **1**, with an apparent p*K*_a_ of 10.6, near the expected p*K*_a_ of 10.71 based on the p*K*_a_ of the propylammonium cation [60,61]. As stated, an obvious third transition point was absent for gelators **2** and **3**. The precipitation or increased turbidity of these gelators at higher pH conditions approaching the theoretical p*K*_a_ of the ammonium cation (Figure S2) could indicate the possibility of another deprotonation at the theoretical p*K*_a_. The lack of a clearly observable transition near 10.7 indicates that most of the ammonium cation protons have been removed in the context of these assembled structures. Parenthetically, we also measured the pH of solutions of gelators **1**, **2**, and **3** in water without any additional base or acid added and found that these solutions had pH values from 5 to 7 (see column 4 of Table 1).

**Figure 4:**
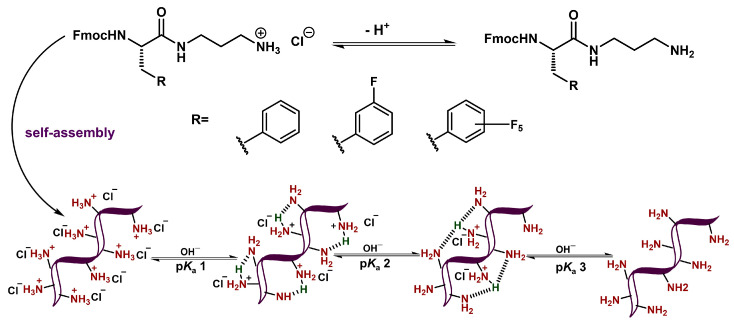
Schematic representation of apparent p*K*_a_ transitions of Fmoc-Phe-DAP ammonium cations in the context of self-assembled materials.

The observation of significantly depressed ammonium p*K*_a_ values (p*K*_a_ 1 and p*K*_a_ 2) can be attributed to the self-assembly of these derivatives as has been observed with other anionic gelators [56,57,59]. When Fmoc-Phe-DAP-derived molecules self-assemble, the ammonium cations are likely in close proximity to those of neighboring molecules within the self-assembled structures (Figure 4). As such, the most acidic p*K*_a_ value (p*K*_a_ 1), which is much lower than the expected theoretical p*K*_a_ of these ammonium protons, likely arises from the reduction in charge–charge repulsion between neighboring ammonium groups when protons are removed. This deprotonation event would leave approximately half the ammonium groups in a cationic charged state, with the other half being in a neutral, deprotonated state. While high-resolution structural data is not yet available for these derivatives, it is logical to assume the polar ammonium groups are solvent-exposed and the observed deprotonation at p*K*_a_ values of ~2.5, much more acidic than the expected values of ~11, is energetically favorable both due to a reduction in charge repulsion effects and to the ability of neighboring deprotonated and protonated amine groups to share the remaining cationic protons (Figure 4). This structural stabilization of the partially protonated state can also help to explain the greater acidity of the protons removed during the second p*K*_a_ transitions (p*K*_a_ 2, 6.6–6.8) relative to the expected acidity of the ammonium cations (~11). The delocalization of the remaining protons increases the acidity for this second transition. Most protons are thus removed well before the pH is basic enough (pH ~11) to remove protons with the expected p*K*_a_ value, thus accounting for the extremely small transition observed at these pH values in the titration experiments. These final transitions may be from the deprotonation of gelator molecules that are not incorporated into the assembled gel network at equilibrium.

### 2.3. Assembly Morphology and Emergent Viscoelastic Properties of Fmoc-Phe-DAP Derivatives

Based on the outcomes of the pH titration experiments, we next sought to examine the self-assembly and emergent viscoelastic properties of these hydrogels at pH values that span the various protonation states. Specifically, gelators **1**, **2**, and **3** were assessed at five different solution pH values: 1.0, 3.0, 5.0, 7.0, and 9.0. These pH values were chosen because they are below the lowest p*K*_a_ transition (pH 1), near the lowest p*K*_a_ transition (p*K*_a_ 1, pH 3), between p*K*_a_ 1 and p*K*_a_ 2 (pH 5), near the second p*K*_a_ transition (p*K*_a_ 2, pH 7), and between p*K*_a_ 2 and theoretical p*K*_a_ (pH 9). pH values above 9.0 were excluded due to gel instability and precipitation (see Figure 2 and Figure S1). Assemblies of compounds **1**, **2**, and **3** were prepared at the target pH values (1, 3, 5, 7, and 9) at 10 mM concentrations of gelator with 114 mM NaCl as described previously. The assemblies of these gelators were analyzed by transmission electron microscopy (TEM) to assess effects of pH on assembly morphology and by oscillatory rheology to determine the impact of pH on the emergent viscoelastic properties of the assembly networks.

**Figure 5:**
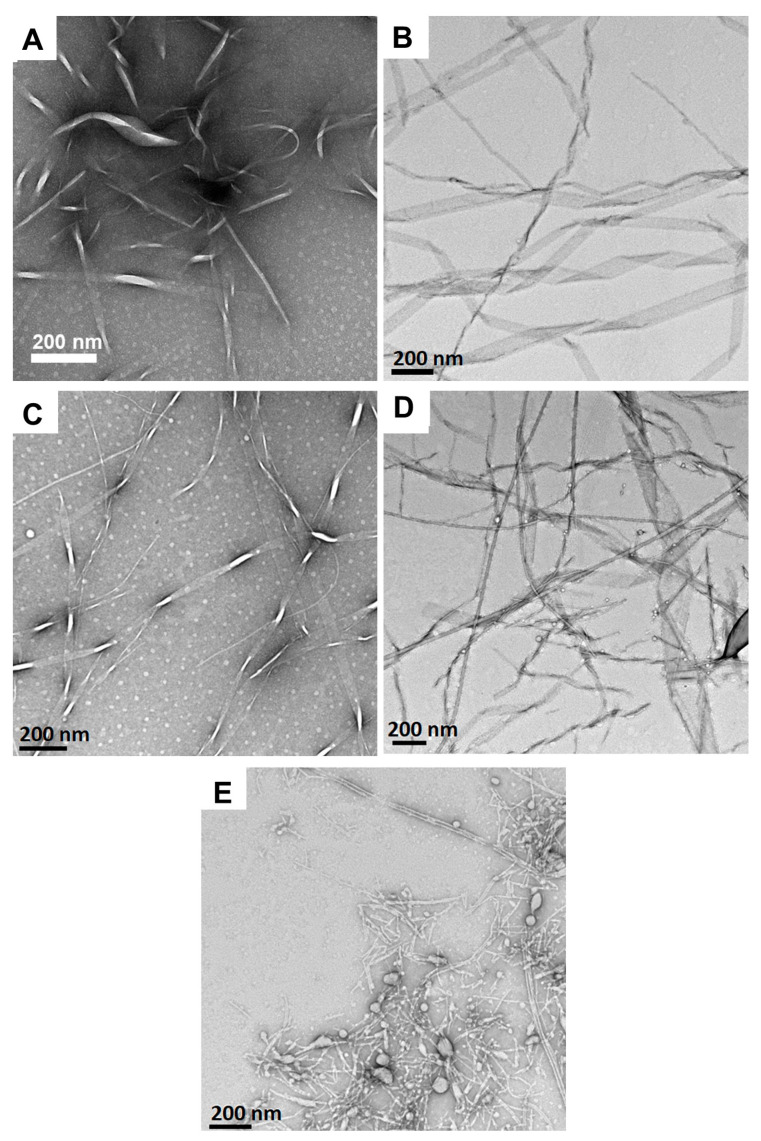
TEM images of hydrogels (10 mM) of Fmoc-Phe-DAP (**1**) at (**A**) pH 1.0, (**B**) pH 3.0, (**C**) pH 5.0, (**D**) pH 7.0, and (**E**) pH 9.0.

We have previously reported that Fmoc-Phe-DAP (**1**) derivatives assemble into polymorphic, sheet-based nanotube structures [53]. Accordingly, we characterized the effect of pH on the assembly morphology of the resulting Fmoc-Phe-DAP hydrogel networks to determine if the pH environment alters these properties. TEM images for Fmoc-Phe-DAP gels at pH values of 1.0, 3.0, 5.0, 7.0, and 9.0 are shown in Figure 5. At pH 1, nanoribbons that range from 17 to 183 nm in diameter were observed (Figure 5A). Interestingly, despite the formation of self-assembled ribbons as observed in TEM images, compound **1** failed to form stable hydrogels (Figure S3). Under these conditions, the pH of the media is less than p*K*_a_ 1. Therefore, the molecules of compound **1** mostly exist in their protonated state. Repulsion between the ammonium cations under these highly acidic conditions (pH less than p*K*_a_ 1) is likely the reason behind collapse of the hydrogel network at this pH. Upon increasing the pH of the media, compound **1** forms stable self-supporting hydrogels (Figure 2). Fibrils and nanoribbons that range from 26 to 262 nm in diameter were observed at pH 3.0, 5.0 and 7.0, like those observed at pH 1.0 (Figure 5B–D). This wide range of polydispersity is observed because images were obtained shortly after initiating self-assembly. These observed morphologies are consistent with previously reported assemblies for Fmoc-Phe-DAP and indicate no significant change in the assembly morphology at acidic and neutral pH. We have previously reported that Fmoc-Phe-DAP assembles into nanotube structures after longer incubation periods (days) [53]. The TEM images herein were obtained after a 24 h incubation time, and the observed nanoribbons are precursors to nanotubes. At pH 5, smaller fibril-like assemblies ranging from 4 to 17 nm in diameter were also observed along with nanoribbons. However, nanoribbons are the more dominant morphology at this pH. These observed morphologies are consistent with previously reported assemblies for Fmoc-Phe-DAP and indicate no significant change in the assembly morphology at acidic and neutral pH. Under these acidic to neutral pH conditions, the ammonium cation is near complete protonation. As such, the similarity in assembly morphology is expected. However, at pH 9.0, an amorphous precipitate (Figure 5E) is observed along with fibrils that were 10.8 ± 3.9 nm in diameter (dimensions are the average of 100 measurements of unique fibrils in ImageJ (version 1.53e) with error reported as the standard deviation). At this more basic pH, which is nearing the expected p*K*_a_ of the ammonium cation, a higher proportion of the ammonium cations are deprotonated. This more highly deprotonated state appears to open unique assembly pathways. In addition, Fmoc-Phe-DAP becomes less soluble when it is deprotonated, likely explaining the appearance of amorphous aggregate.

**Figure 6:**
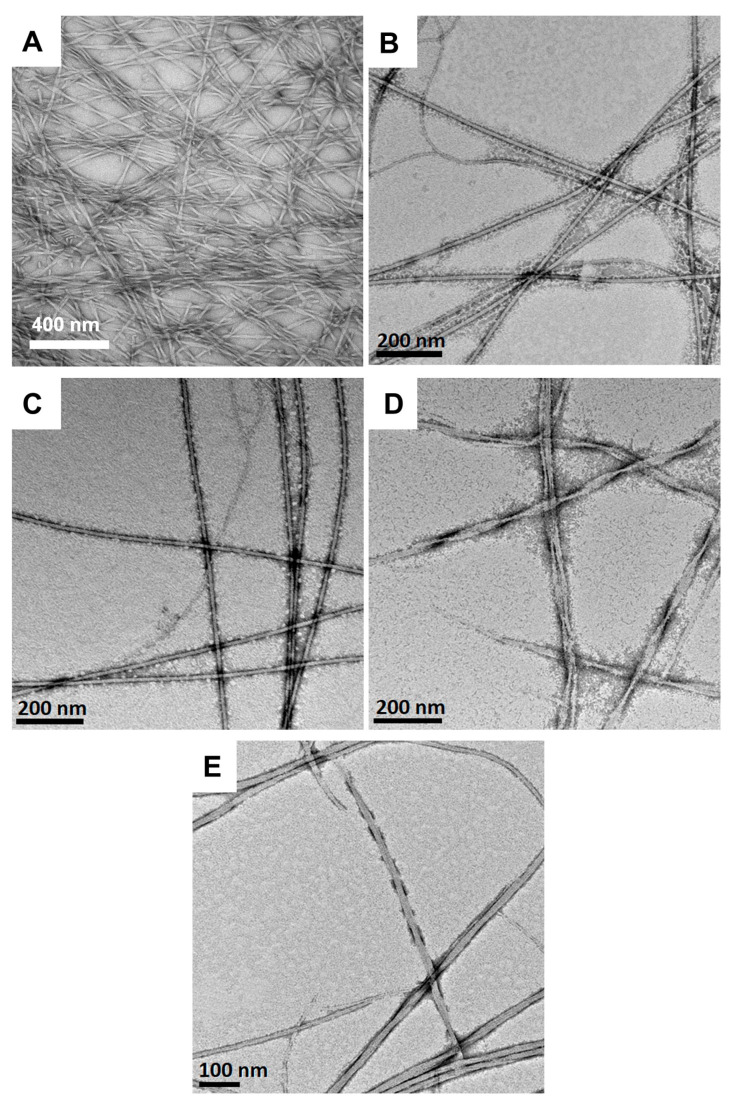
TEM images of hydrogels (10 mM) of Fmoc-3F-Phe-DAP (**2**) at (**A**) pH 1.0, (**B**) pH 3.0, (**C**) pH 5.0, (**D**) pH 7.0, and (**E**) pH 9.0.

The morphology of Fmoc-3F-Phe-DAP (**2**) assemblies that form observed hydrogel networks was also assessed at pH 1.0, 3.0, 5.0, 7.0, and 9.0. Assemblies of **2** exhibited morphologies that are distinct from those observed for gelator **1**. While the Fmoc-Phe-DAP (**1**) assemblies were primarily twisted nanoribbon structures of various dimensions, the assemblies of the fluorinated Fmoc-3F-Phe-DAP (**2**) were instead more uniform fibril-like assemblies (Figure 6). At pH 1.0, twisted fibers that measure 20.6 ± 6.7 nm in diameter are observed (Figure 6A). Similarly to compound **1**, the assemblies of compound **2** fail to form a stable hydrogel network at this pH (Figure S3), again likely due to extensive charge repulsion effects in the fully protonated state. The fibrils observed at pH 3.0 and 5.0 (Figure 6B,C) are visually indistinguishable, but measure 10.1 ± 2.6 nm in diameter at pH 3.0 and 19.7 ± 5.9 nm in diameter at pH 5.0. Similar fibers are also observed at pH 7.0 and 9.0, although twisted fibers are also observed at these more basic pH conditions (Figure 6D,E). These twisted ribbons are 18.7 ± 7.4 nm in diameter at pH 7 and 19.2 ± 7.9 nm in diameter at pH 9. These subtle morphological differences in the assemblies may account for the observed transparency of the gels: gels at pH 3.0 and 5.0 are more transparent, while gels at pH 7.0 and 9.0 are more opaque. The observed assemblies formed stable hydrogel networks between pH 3.0 and 9.0. TEM images corroborate that this is likely because the structure of the network assemblies does not vary with pH, with no amorphous precipitates detected as was observed at high pH with Fmoc-Phe-DAP (**1**).

**Figure 7:**
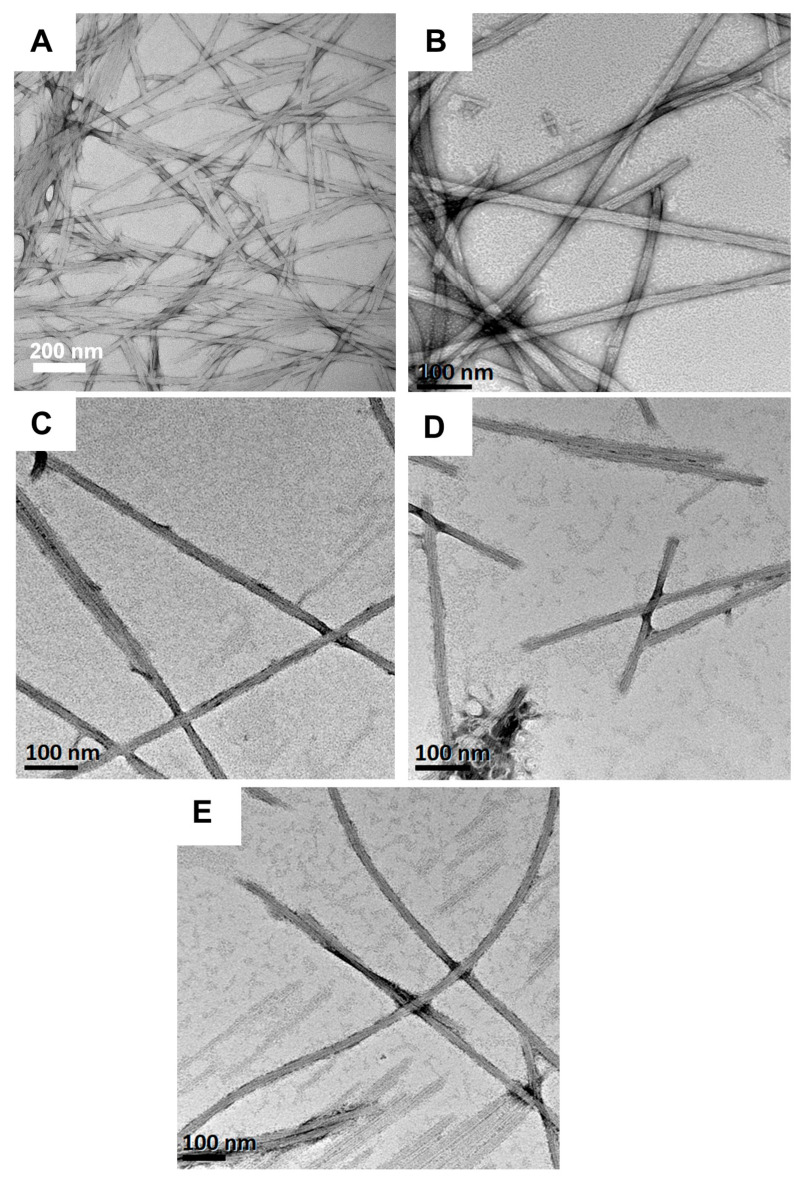
TEM images of hydrogels (10 mM) of Fmoc-F_5_-Phe-DAP (**3**) at (**A**) pH 1.0, (**B**) pH 3.0, (**C**) pH 5.0, (**D**) pH 7.0, and (**E**) pH 9.0.

Lastly, the morphologies of Fmoc-F_5_-Phe-DAP (**3**) hydrogel assemblies were characterized across the various pH conditions. TEM images show very similar nanofibers from acidic pH 1.0 to basic pH 9.0 (Figure 7). We have previously found hydrogels of this pentafluorinated Phe derivative are the most stable to environmental and temporal changes [55,62]. These hydrogels also show strong uniformity in assembly morphology across different pH conditions. The observed fibrils are 19.4 ± 4.6 nm in diameter at pH 1, 21.8 ± 5 nm in diameter at pH 3, 18.9 ± 8.1 nm in diameter at pH 5, 14.4 ± 3.8 nm in diameter at pH 7, and 21.2 ± 8.7 nm in diameter at pH 9 (Figure 7A–E). However, at pH **1** (less than p*K*_a_ 1) gelator **3** failed to form a self-supporting hydrogel (Figure S3). Again, we suspect this is due to charge repulsion effects that destabilize network formation that provides stable hydrogels. As we have previously observed, the morphology of self-assembly of Fmoc-F_5_-Phe-DAP is less sensitive to environmental conditions than other Phe derivatives that have been studied. The effect of perfluorination of the gelator side chain seems to dominate the observed assembly pathways [55].

**Figure 8:**
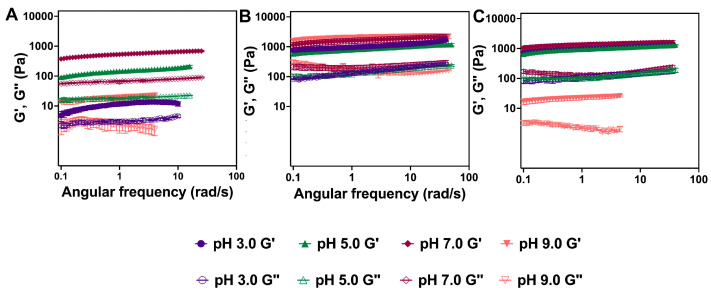
Representative frequency sweep data collected via oscillatory rheology for 10 mM hydrogels of (**A**) Fmoc-Phe-DAP (**1**), (**B**) Fmoc-3F-Phe-DAP (**2**), and (**C**) Fmoc-F_5_-Phe-DAP (**3**). G′ and G″ values (Pa) are represented by closed shapes and open shapes, respectively. Plots for each individual experiment at each pH are shown in the Supporting Information (Figures S5–S7).

Oscillatory rheology reveals significant differences in the viscoelastic properties of the hydrogels of these derivatives as a function of pH. The viscoelastic properties of the hydrogels were characterized at pH 3.0, 5.0, 7.0, and 9.0. The viscoelastic properties of the assemblies were not characterized at pH 1 since the hydrogel networks collapse at this pH (Figure S3). Specifically, we characterized the storage and loss moduli (G′ and G″, respectively) using oscillatory rheology frequency sweep experiments (Figure 8) at a constant strain of 0.2% which is within the viscoelastic region of the hydrogels (Figure S4). We first determined the storage and loss moduli of Fmoc-Phe-DAP (**1**) hydrogels as a function of pH (Figure 8A and Figure S5). Even though the network structure seemed very similar by TEM, Fmoc-Phe-DAP gels are substantially stronger at pH 7.0 (G′: 604.7 Pa), weaker at pH 5.0 (G′: 150 Pa), and very weak at pH 3.0 (G′: 12.3 Pa), and 9.0 (G′: 10.6 Pa) (Table 2). For all gels, the G′ (the solid-like property of the gel) was higher than G″ (the liquid-like property of the gel), and were parallel and separated by roughly an order of magnitude, indicating a structurally robust hydrogel state [63]. These results indicate that while pH does not strongly impact the morphology of the assemblies as determined by TEM, pH does impact the network structure and the viscoelastic properties of the networks.

Hydrogels of Fmoc-3F-Phe-DAP (**2**) had unique viscoelastic properties compared to hydrogels of Fmoc-Phe-DAP (**1**). The rheological viscoelasticity of Fmoc-3F-Phe-DAP (**2**) hydrogels (Figure 8B and Figure S6) showed similar G’ values for the gels at pH 3.0 and 5.0 (1172 Pa and 914 Pa, respectively) (Table 2), which correlates with the network structure and morphological appearance. The rheological G′ values for the gels at pH 7.0 and 9.0 are slightly higher (1684 Pa and 2085 Pa, respectively), which also correlates with the twisted assembly morphological structure and more opaque physical appearance. With this gelator, also G′ values were higher than G″ values by approximately an order of magnitude, indicating the desired hydrogel state [63]. The observed differences in emergent viscoelasticity for Fmoc-3F-Phe-DAP hydrogels are only subtly different across these pH ranges, indicating that fluorination of the side chain produces networks that are less sensitive to differences in pH than the nonfluorinated Fmoc-Phe-DAP derivative.

Finally, the emergent viscoelasticity of Fmoc-F_5_-Phe-DAP (**3**) hydrogels was characterized. The viscoelasticity of these hydrogels is similar at pH 3.0, 5.0, and 7.0 (G′: 1103 Pa, 1228 Pa, and 1423 Pa, respectively) (Figure 8C and Figure S7), consistent with the similar morphology of the assemblies under these pH conditions. Hydrogels of Fmoc-F_5_-Phe-DAP become weaker and more opaque at pH 9.0 (G′ 26 Pa), even though the assembly morphology does not appear significantly different at this pH compared to the other pH conditions. The observed G′ values were higher than G″ values by approximately an order of magnitude, indicating a structurally robust hydrogel state [63]. There are no significant differences in the viscoelastic properties of Fmoc-F_5_-Phe-DAP at pH 3, like Fmoc-3F-Phe-DAP. Again, this reinforces the conclusion that fluorination of the attached aromatic ring helps to stabilize the assembly and network formation of this derivative independent of environment.

For all the gelators, we found some significant differences in the network structure and/or mechanical properties of the gels when the pH was changed from 3.0 to 9.0. These cationic gelators are sensitive to solution ionic strength and cation identity to form gels [55,62], but the specific impacts are correlated with the degree of fluorination of the aromatic side chain. The nonfluorinated Fmoc-Phe-DAP (**1**) gelator is more susceptible to changes in pH, in that it forms the strongest gels at pH 7.0 but weaker hydrogels at more acidic pH (pH 3.0 and 5.0) and at basic pH (pH 9.0). As shown in TEM images (Figure 5E), deprotonation of the ammonium cation at pH 9.0 disfavors assembly of **1** into network-forming nanoribbons and fibrils, instead promoting the formation of amorphous aggregates, thereby accounting for the dramatic loss of viscoelasticity. The emergent viscoelasticity of Fmoc-3F-Phe-DAP (**2**) and Fmoc-F_5_-Phe-DAP (**3**) hydrogels is greater than that of Fmoc-Phe-DAP (**1**) hydrogels, with storage moduli values an order of magnitude greater or more under almost all pH conditions. In addition, the viscoelasticity of Fmoc-3F-Phe-DAP (**2**) and Fmoc-F_5_-Phe-DAP (**3**) hydrogels is less sensitive to changes in pH than Fmoc-Phe-DAP (**1**) hydrogels. Hydrogels of Fmoc-3F-Phe-DAP (**2**) exhibited storage moduli of at least 1000 Pa at all pH values assessed. Hydrogels of compound **3** also had storage moduli of at least 1000 Pa at all pH values except at pH 9.0, where the storage modulus dropped dramatically to only 26 Pa. Fmoc-3F-Phe-DAP (**2**) is significantly more soluble than Fmoc-F_5_-Phe-DAP (**3**) due to the increased hydrophobicity of the pentafluorinated gelator **3** [53]. Thus, this significant drop in storage modulus of hydrogels of **3** at pH 9.0 is likely due to partial collapse and precipitation of the hydrogel network that occurs because of the increased hydrophobicity of Fmoc-F_5_-Phe-DAP (**3**) upon more complete deprotonation at this pH. The decreased hydrophobicity of Fmoc-3F-Phe-DAP (**2**) allows its hydrogels to avoid a similar collapse at pH 9.0. Monofluorination of Fmoc-3F-Phe-DAP (**2**) better balances hydrophobic and aromatic π-π effects that stabilize the self-assembled hydrogel networks. While detailed structural characterization of the packing order of these derivatives is currently unavailable, we presume that monofluorination stabilizes aromatic π-π effects in a manner similar to that previously reported for parent Fmoc-Phe carboxylic acid derivatives [64]. These rheological experiments reinforce that fluorination of the phenylalanine side chain group makes the resulting hydrogels less susceptible to the pH of the media, allowing these materials to be used for applications across a broader pH range than nonfluorinated Fmoc-Phe-DAP (**1**) hydrogels.

## 3. Conclusions

We have demonstrated the variation in gelation capability, network structure, and viscoelastic properties of cationic Fmoc-Phe-derived gelators as a function of environmental pH. It was observed that the self-assembly properties of these derivatives result in modification of the specifically observed p*K*_a_ values, with several deprotonation events occurring at lower p*K*_a_ than the expected values of ~11. These more acidic deprotonation states arise to relieve charge repulsion during the structural transitions that occur during the self-assembly process. Further, these shifted p*K*_a_ values do not significantly differ between the gelators, with the fluorinated derivatives (**2** and **3**) exhibiting similar p*K*_a_ values to the nonfluorinated Fmoc-Phe-DAP derivative (**1**) despite the presence of electronegative fluorine atoms. This could be attributed to the large distances between the fluorinated groups and the ammonium cation moieties, which limits the influence of the electron deficient benzene groups on ammonium ion acidity. We have also demonstrated that the degree of side chain modification decreases the susceptibility of the gelators to changes in fibril morphology and emergent viscoelastic properties as a function of environmental pH. When the gelator is not fluorinated (gelator **1**), the emergent hydrogel viscoelasticity is much more sensitive to changes in pH. In contrast, for fluorinated gelators, the properties of these hydrogels are less susceptible to the specific pH of the media.

These results demonstrate that pH can be used to modify the emergent properties of Fmoc-Phe-DAP-derived supramolecular hydrogels to varying degrees depending on the structure of the gelator. These properties are possibly relevant to various applications. For example, fluorinated Fmoc-Phe-DAP derivatives (gelators **2** and **3**) are attractive for applications in which hydrogels that retain stability across a broad range of pH conditions are required such as localized, sustained release of therapeutics over longer time periods under conditions where changes in local pH may occur as a function of specific physiological events. In contrast, gelator **1**, which exhibits viscoelastic properties that are more sensitive to differences in pH conditions, has potential for applications in which the evolution of hydrogel properties in response to changes in pH is desirable. For example, selective release of therapeutics in tumor microenvironments, which are often more acidic than typical tissues, may be possible with Fmoc-Phe-DAP hydrogels as they collapse at acidic pH. These outcomes provide important insight into the influence of pH on the self-assembly and correlating emergent properties of supramolecular hydrogel networks and provide design cues for the formulation of hydrogels for applications in which varying pH conditions may exist.

## 4. Materials and Methods

### 4.1. Materials

Reagents and organic solvents were purchased commercially and used without further purification. Compounds **1**–**3** were synthesized by previously reported protocols [54]. Water used for gelation and preparation of solutions was purified using a nanopure filtration system (Barnstead NANOpure (Thermo Scientific, Waltham, MA, USA), 0.2 μm filter, 18.2 MΩ cm).

### 4.2. Self-Assembly Conditions

All compounds were assembled with a final gelator concentration of 10 mM, final NaCl concentration of 114 mM, and total volume of 2 mL. Compounds **1**–**3** (0.02 mmol) were dissolved in 1.5 mL of water at 70 °C in a glass vial, followed by sonication until complete dissolution was observed. The pH was adjusted using 0.1 M NaOH and/or 0.1 M HCl (0.5 M HCl to attain pH 1) solutions until the desired pH was reached (pH measured using 6.5 mm diameter pH probe, VWR sympHony, 89231-592, VWR, Radnor, PA, USA). Then, 0.4 mL of a NaCl solution (570 mM) was added to the vial, which was immediately and briefly agitated by a vortex mixer. The samples were allowed to stand for 24 h. Formation of a self-supporting hydrogel was determined by a vial inversion test. Digital images of all assemblies at 24 h after salt addition are provided (Figures S1 and S2, Supporting Information and figures provided herein).

### 4.3. Determination of the Apparent pK_a_ by Titration

A 6.5 mm diameter pH probe (VWR symphony, 89231-592) was employed for all pH measurements. Compounds 1–3 were dissolved in 10 mL of nanopure water adjusted to pH ~2.0 with a 0.1 M HCl solution to a final concentration of 1 mM. The p*K*_a_ values were determined by titration via the addition of aliquots of a 0.1 M NaOH solution. pH values were recorded until reaching a stable value after each addition during the titration process. The solutions were gently stirred to prevent the formation of gel or precipitate. The p*K*_a_ values were obtained using the Henderson–Hasselbalch equation (Equation (1)) [65].

pH = p*K*_a_ + log([A^−^]/[HA]) (1)

The [A^−^] term refers to the concentration of the conjugate base, and [HA] is the concentration of the weak acid. Therefore, p*K*_a_ is equal to the pH at the half neutralization point. The second derivative method was used to precisely determine the volume of base corresponding to the equivalence point. The intercept after each maximum of the second derivative versus volume plots (Figure 3) gives the volume of base of the equivalence point for each p*K*_a_ transition. The p*K*_a_ values were determined by correlation of the equivalence volume to the pH versus volume plot. The p*K*_a_ is the volume corresponding to the half-neutralization points on the pH versus volume plots [66]. The second derivative and pH vs. volume curves were plotted using GraphPad Prism (version 10.6.1, San Diego, CA, USA). The data was analyzed using Excel and GraphPad Prism.

### 4.4. Transmission Electron Microscopy (TEM)

Negative stain TEM images were obtained to define the morphology of the self-assembled structures that define the hydrogel network. A Hitachi 7650 transmission electron microscope (Hitachi High-Tech America, Hillsboro, OR, USA) with an accelerating voltage of 80 kV was used to obtain all negative stain TEM images. Sample preparation was completed by applying aliquots of assembled materials (5 μL) directly onto 200 mesh carbon-coated copper grids and allowing the sample to stand for 1 min. Excess liquid sample was carefully removed by capillary action using filter paper. Each grid with deposited sample was then stained with 2% (*w*/*v*) uranyl acetate (5 μL) for 10 min. Excess stain solution was then removed by capillary action and the grids were allowed to air-dry for at least 5 min prior to imaging.

### 4.5. Oscillatory Rheology

The viscoelasticity of all hydrogels was characterized by oscillatory rheology. These experiments were conducted with a TA Instruments Discovery HR-2 rheometer (New Castle, DE, USA) operating in oscillatory mode. Experiments were performed using a 20 mM parallel plate geometry and a standard Peltier plate. Hydrogels (1 mL) were formed in 1.5 mL plastic microcentrifuge tubes according to the assembly procedure described in the “Self-Assembly Conditions” section above. All gels were allowed to mature for 24 h before rheological analysis. Experiments were set with an average gap size of 1.4 mm. Strain sweep experiments were performed from 0.01 to 100% strain at a constant angular frequency of 1 Hz (6.28 rad/s) to determine the linear viscoelastic region for each sample (Figure S4, Supporting Information). Prior to frequency sweep measurements, a time sweep at constant strain of 0.2% and constant angular frequency of 0.1 rad/s was performed over 300 s to allow the sample to equilibrate on the plate after the transferal of the hydrogel sample from the microfuge tube to the Peltier plate. Frequency sweep experiments were then performed for each sample from 0.1 to 100 rad/s at a constant strain of 0.2%, which was within the linear viscoelastic region for all hydrogels studied. Values at the upper end of the frequency sweep were eliminated from reported data when the raw phase angle increased above 175° as recommended for the TA DHR series of rheometers. Raw phase angle values beyond 175° are dominated by instrument inertial torque instead of the sample torque [67]. Reported values for storage moduli (G′) and loss moduli (G″) are the average of three distinct measurements on separate hydrogels with the error reported as the standard deviation about the mean.

## Figures and Tables

**Table 1:** Experimental p*K*_a_ values obtained from titration curves for gelators **1**, **2**, and **3** and the pH of solutions of each gelator dissolved in water without any additional acid or base added.

Gelator	p*K*_a_ 1	p*K*_a_ 2	p*K*_a_ 3	pH in Water
Fmoc-Phe-DAP (**1**)	2.46	6.82	10.6	7.0 ± 0.04
Fmoc-3F-Phe-DAP (**2**)	2.47	6.65	–	5.8 ± 0.05
Fmoc-F_5_-Phe-DAP (**3**)	2.53	6.78	–	5.0 ± 0.05

**Table 2:** Average G′ and G″ values for each hydrogel determined from frequency sweep experiments shown in Figure 8. Data is in Pa units. Error is reported as the standard deviation.

	Gelator		
pH	Fmoc-Phe-DAP (1) (Pa)	Fmoc-3F-Phe-DAP (2) (Pa)	Fmoc-F_5_-Phe-DAP (3) (Pa)
3.0	G′: 12.3 ± 2.8 G″: 3.3 ± 0.4	G′: 1172.6 ± 264.6 G″: 180.9 ± 67.6	G′: 1103.3 ± 176.3 G″: 146.1 ± 37.2
5.0	G′: 150.0 ± 27.3 G″: 18.9 ± 1.7	G′: 1057.9 ± 287.9 G″: 173.2 ± 52.8	G′: 1228.3 ± 199.5 G″: 154.5 ± 37.8
7.0	G′: 604.7 ± 111.8 G″: 75.2 ± 5.9	G′: 1684.4 ± 397.1 G″: 221.3 ± 47.7	G′: 1423.0 ± 172.6 G″: 166.3 ± 37.8
9.0	G′: 10.6 ± 2.9 G″: 2.3 ± 0.5	G′: 2085.1 ± 319.7 G″: 171.8 ± 51.9	G′: 26.0 ± 2.4 G″: 2.8 ± 0.7

## Data Availability

The data presented in this study are available upon request from the corresponding author.

## Supplementary Materials

The following are available online at https://www.mdpi.com/article/10.3390/gels11110877/s1, Figure S1: Digital images of hydrogels at pH 3–10 after 2 weeks of incubation. Figure S2: Digital images of hydrogels at pH 2 and pH 12 (before and after titration with NaOH). Figure S3: Digital images of suspensions/solutions of gelators at pH 1.0. Figure S4: Rheological strain sweep data used to determine the linear viscoelastic region for hydrogels studied herein. Figure S5: Individual rheology plots from Figure 8A. Figure S6: Individual rheology plots from Figure 8B. Figure S7: Individual rheology plots from Figure 8C.

## Abbreviations

The following abbreviations are used in this manuscript:

DAP	diaminopropane
Fmoc	fluorenylmethoxycarbonyl
LMW	low molecular weight
Phe	phenylalanine
TEM	transmission electron microscopy
