# Supplementary material for: Effect of pH on the Emergent Viscoelastic Properties of Cationic Phenylalanine-Derived Supramolecular Hydrogels
Source: Gels. 2025 Nov 1;11(11):877. doi: 10.3390/gels11110877 (PMC12652706; doi:10.3390/gels11110877)
Supplement: Supplementary file 1 [file gels-11-00877-s001.zip › gels-3905125-supplementary.pdf]

Effect of pH on the Emergent Viscoelastic Properties of Cationic Phenylalanine-Derived Supramolecular Hydrogels

*Pamela Agredo,[a] Shruti Ghosh,[a] Brittany L. Abraham,[a] and Bradley L. Nilsson[a,b]**

[a]Department of Chemistry, University of Rochester, Rochester, NY, 14627-0216, USA

[b]Materials Science Program, University of Rochester, Rochester, NY 14627-0166, USA

E-mail: bradley.nilsson@rochester.edu

Tel. +1 585 276-3053

SUPPORTING INFORMATION

Contents:

Figure S1. Digital images of A) Fmoc-Phe-DAP (gelator 1), B) Fmoc-3F-Phe-DAP (gelator 2), and C) Fmoc-F5-Phe-DAP (gelator 3) gels at different pH values, from 3.0 to 10.0 (left to right), after 2 weeks. 2
Figure S2. Digital images of solutions/suspensions of gelators 1, 2, and 3. A) Solutions (1 mM) of gelators 1, 2, and 3 (from left to right) before titration (pH 2.0), and B) Solutions (1 mM) of gelators 1, 2, and 3 (from left to right) after titration (pH 12.0). 3
Figure S3. Digital images showing suspensions of Fmoc-Phe-DAP (gelator 1), Fmoc-3F-Phe-DAP (gelator 2) and Fmoc-F_5-Phe-DAP (gelator 3) at pH 1. 3
Figure S4. Strain sweep data collected via oscillatory rheology of 10 mM hydrogels of A) Fmoc-Phe-DAP (**1**), B) Fmoc-3F-Phe-DAP (**2**), and C) Fmoc-F_5-Phe-DAP (**3**) at pH 3.0, 5.0, 7.0, and 9.0. 4
Figure S5. Oscillatory rheology frequency sweep plots of Fmoc-Phe-DAP (**1**) hydrogels at A) pH 3.0, B) pH 5.0, C) pH 7.0, and D) pH 9.0. 4
Figure S6. Oscillatory rheology frequency sweep plots of Fmoc-3F-Phe-DAP (**2**) hydrogels at A) pH 3.0, B) pH 5.0, C) pH 7.0, and D) pH 9.0. 5
Figure S7. Oscillatory rheology frequency sweep plots of Fmoc-F_5-Phe-DAP (**3**) hydrogels at A) pH 3.0, B) pH 5.0, C) pH 7.0, and D) pH 9.0. 6

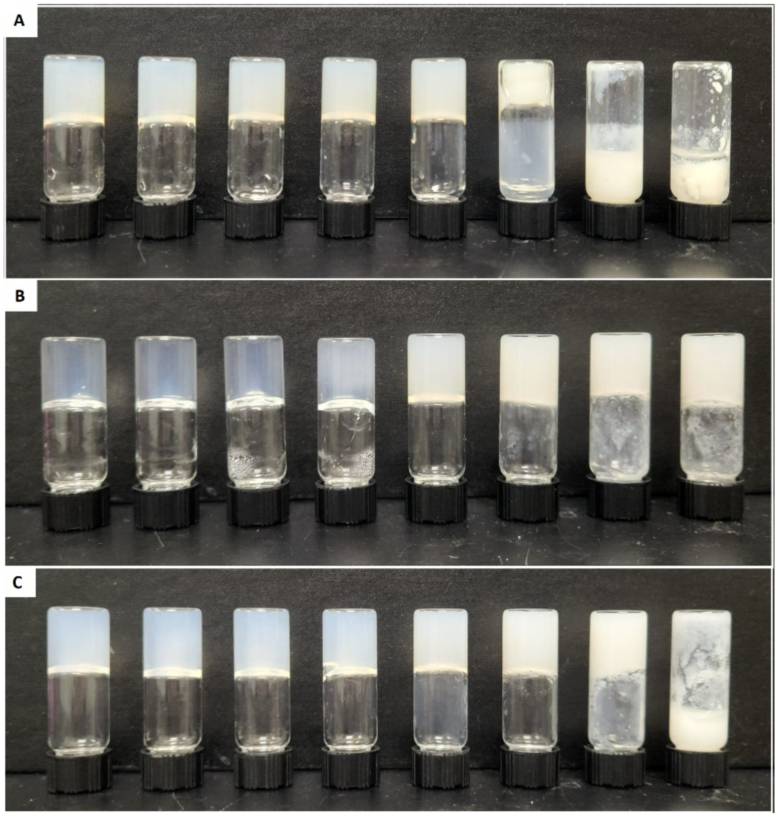

Figure S1. Digital images of A) Fmoc-Phe-DAP (gelator **1**), B) Fmoc-3F-Phe-DAP (gelator **2**), and C) Fmoc-F5-Phe-DAP (gelator **3**) gels at different pH values, from 3.0 to 10.0 (left to right), after 2 weeks.

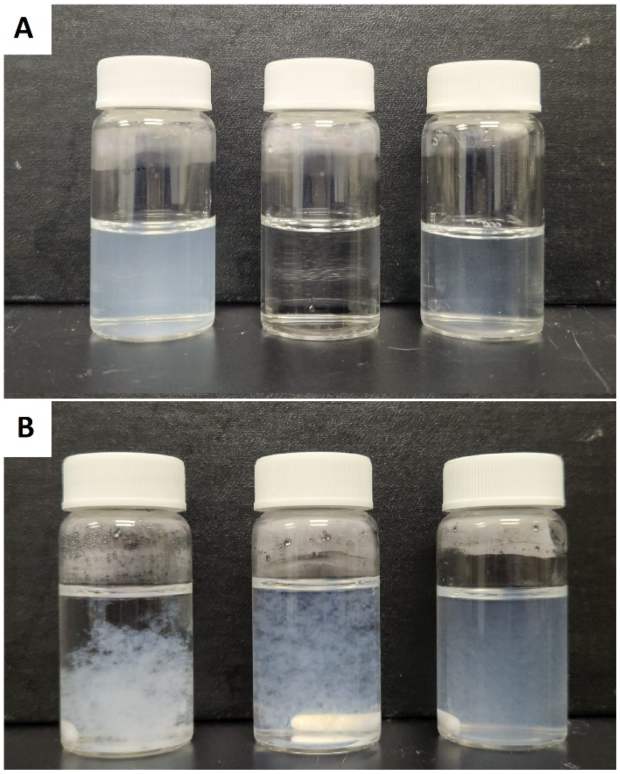

Figure S2. Digital images of solutions/suspensions of gelators 1, 2, and 3. A) Solutions (1 mM) of gelators 1, 2, and 3 (from left to right) before titration (pH 2.0), and B) Solutions (1 mM) of gelators 1, 2, and 3 (from left to right) after titration (pH 12.0).

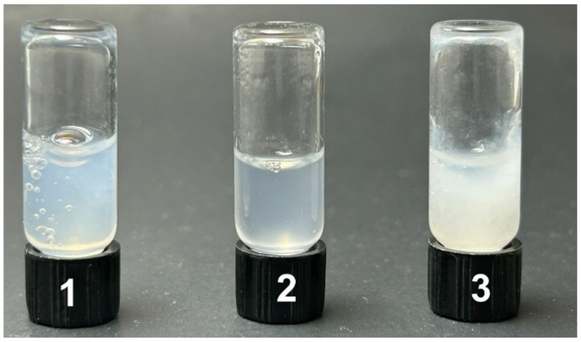

Figure S3. Digital images showing suspensions of Fmoc-Phe-DAP (gelator 1), Fmoc-3F-Phe-DAP (gelator 2) and Fmoc-F_5-Phe-DAP (gelator 3) at pH 1.

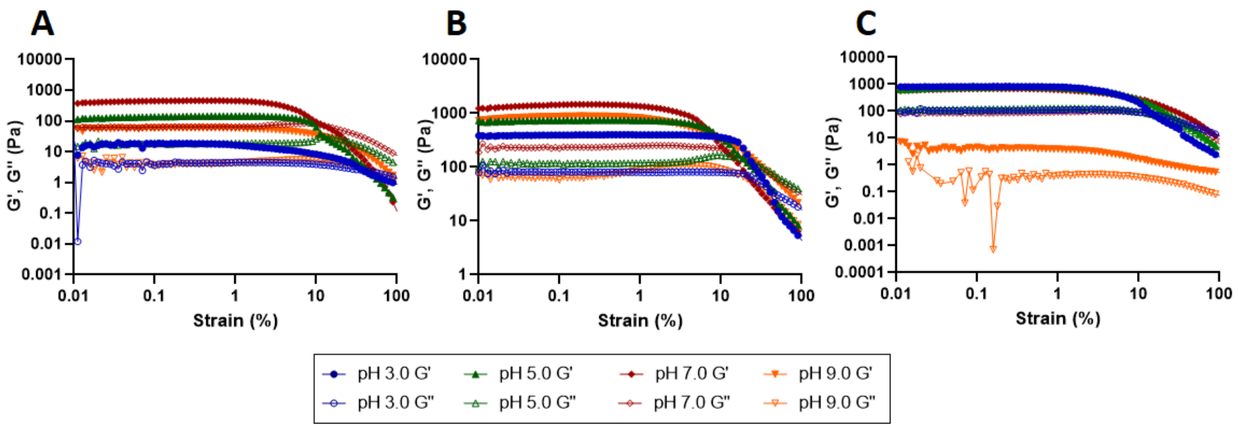

Figure S4. Strain sweep data collected via oscillatory rheology of 10 mM hydrogels of A) Fmoc-Phe-DAP (**1**), B) Fmoc-3F-Phe-DAP (**2**), and C) Fmoc-F_5-Phe-DAP (**3**) at pH 3.0, 5.0, 7.0, and 9.0.

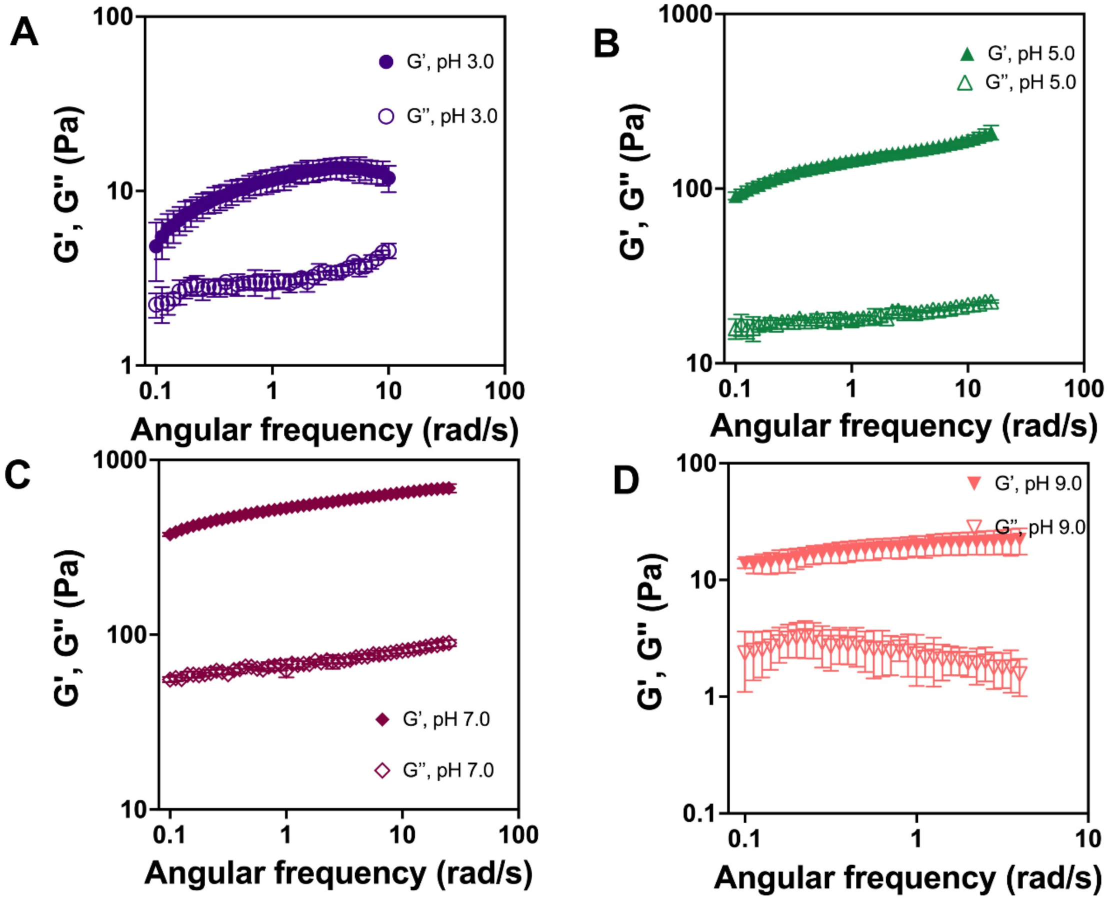

Figure S5. Oscillatory rheology frequency sweep plots of Fmoc-Phe-DAP (**1**) hydrogels at A) pH 3.0, B) pH 5.0, C) pH 7.0, and D) pH 9.0.

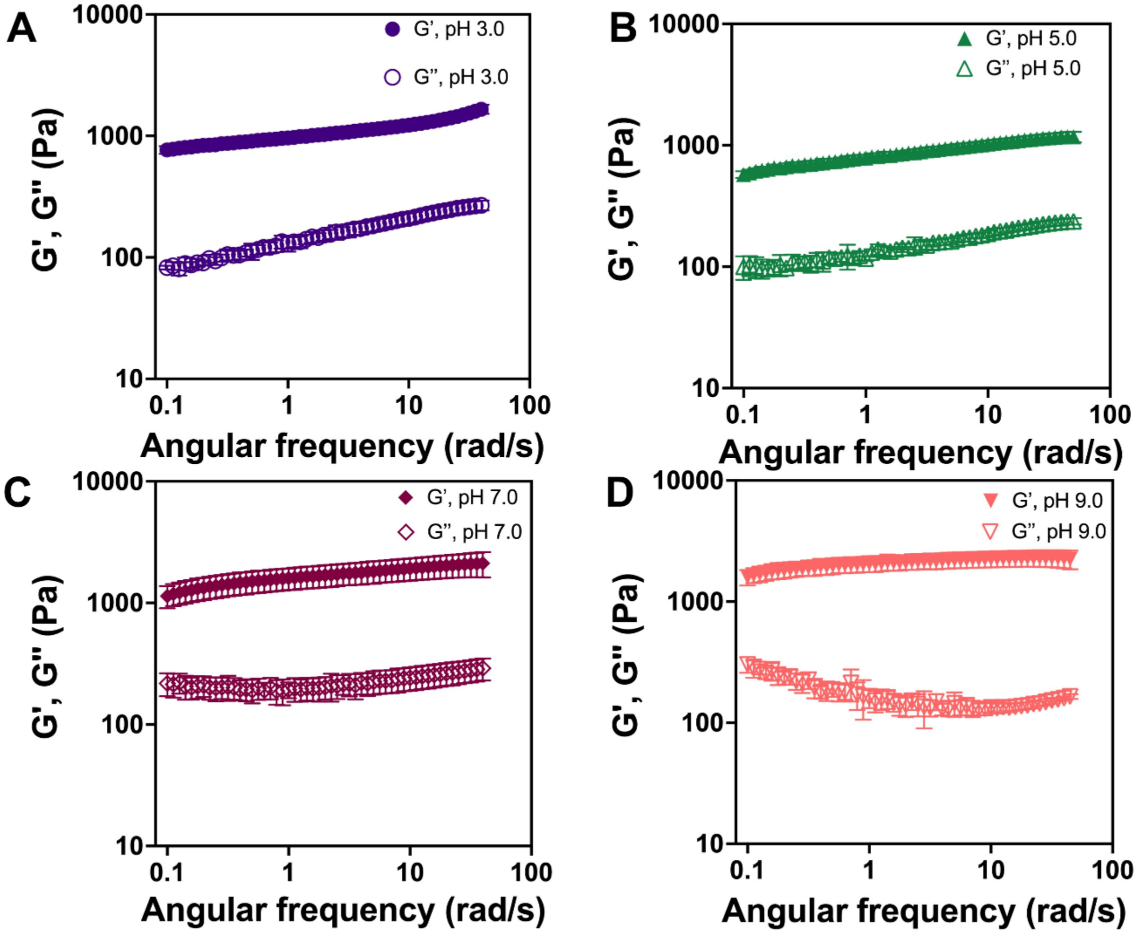

Figure S6. Oscillatory rheology frequency sweep plots of Fmoc-3F-Phe-DAP (**2**) hydrogels at A) pH 3.0, B) pH 5.0, C) pH 7.0, and D) pH 9.0.

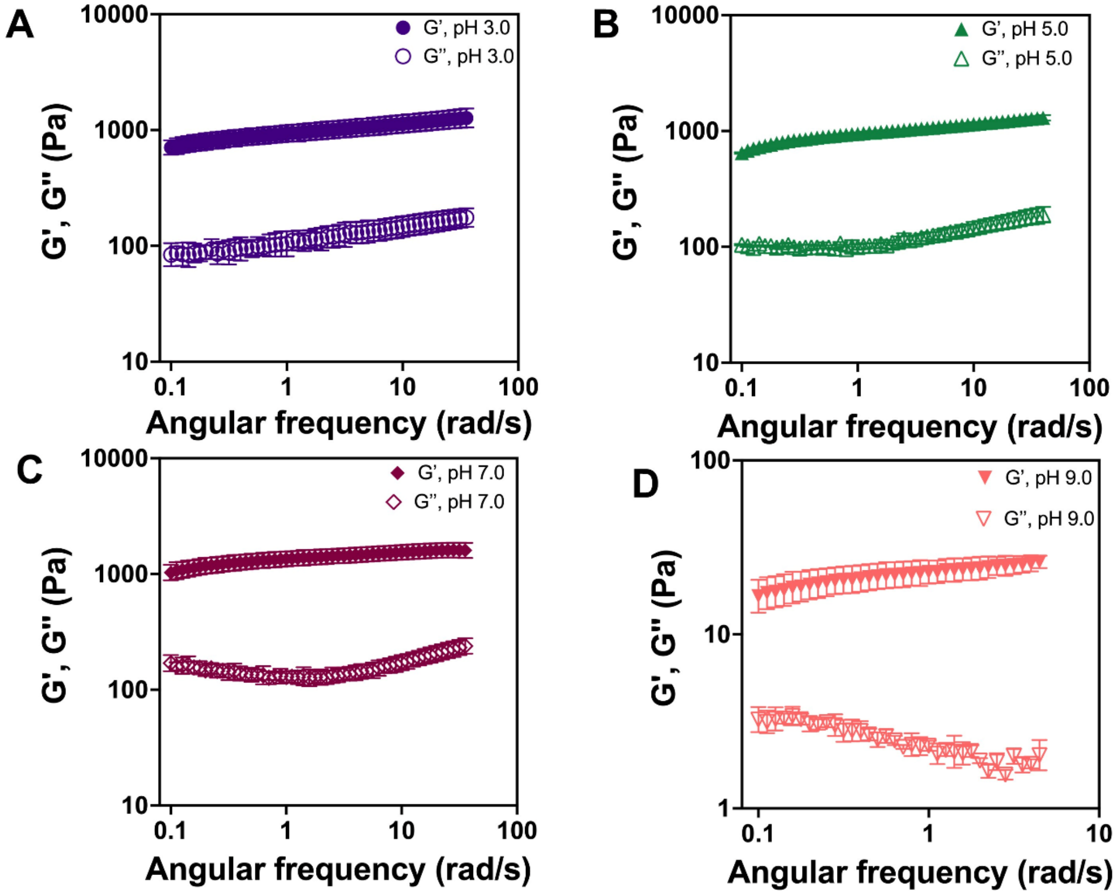

Figure S7. Oscillatory rheology frequency sweep plots of Fmoc-F_5-Phe-DAP (**3**) hydrogels at A) pH 3.0, B) pH 5.0, C) pH 7.0, and D) pH 9.0.
